# Effect of different home-cooking methods on the bioaccessibility of zinc and iron in conventionally bred cowpea (*Vigna unguiculata* L. Walp) consumed in Brazil

**DOI:** 10.3402/fnr.v60.29082

**Published:** 2016-03-03

**Authors:** Elenilda J. Pereira, Lucia M. J. Carvalho, Gisela M. Dellamora-Ortiz, Flávio S. N. Cardoso, José L. V. Carvalho

**Affiliations:** 1 Department of Natural Products and Food, Universidade Federal do Rio de Janeiro, Rio de Janeiro, Brazil; 2 School of Pharmacy, Universidade Federal do Rio de Janeiro, Rio de Janeiro, Brazil; 3 Embrapa Food Technology, Rio de Janeiro, Brazil

**Keywords:** bioaccessibility, iron, zinc, *Vigna unguiculata*, home-cooking methods

## Abstract

**Background:**

The cowpea (*Vigna unguiculata* L. Wap.) is an excellent source of iron and zinc. However, iron from plant sources is poorly absorbed compared with iron from animal sources.

**Objectives:**

The objective of this study was to evaluate iron and zinc bioaccessibility in cowpea cultivars after processing.

**Methods:**

Zinc and iron bioaccessibilities in cowpea samples were determined based on an *in vitro* method involving simulated gastrointestinal digestion with suitable modifications.

**Results:**

When water-soaked beans were cooked in a regular pan, the highest percentage of bioaccessible iron obtained was 8.92%, whereas when they were cooked in a pressure cooker without previous soaking, the highest percentage was 44.33%. Also, the percentage of bioaccessible zinc was 52.78% when they were cooked in a regular pan without prior soaking. Higher percentages of bioaccessible iron were found when cooking was done in a pressure cooker compared with regular pan cooking. In all cultivars, cooking of cowpea beans in both pressure cooker and in a regular pan yielded higher percentages of bioaccessible zinc compared with availability of bioaccessible iron.

**Conclusions:**

Iron bioaccessibility values suggest that cooking in a regular pan did not have a good effect on iron availability, since the percentage of bioaccessible iron was lower than that of zinc. The determination of iron and zinc bioaccessibility makes it possible to find out the actual percentage of absorption of such minerals and allows the development of efficient strategies for low-income groups to access foods with high levels of these micronutrients.

Deficiencies of micronutrients such as iron and zinc affect thousands of needy people around the world, especially in developing countries (1–3). In Brazil, iron deficiency anemia is a nutritional problem of great magnitude and injury, especially in children aged under 2 years and pregnant women, affecting about 50 and 35% of these two population groups, respectively (4).

Iron deficiency is not only caused by low dietary intake but also by many factors that affect its bioavailability in food (5). Beans are the second most consumed staple food in Brazil, and part of the daily menu of most of the population, providing important nutrients in the human diet, such as protein, iron, zinc, and vitamins. Beans are also the best vegetable source of iron (5.3–8.5 mg/100 g) and therefore an ally in combating nutritional deficiencies. Although beans are considered the best plant source of iron, the total amount of the nutrient is not fully bioavailable (6, 7).

Among the legumes, the cowpea beans (*Vigna unguiculata* (L.) Wap.) are one of the most important staple foods for people in north and northeast Brazil, for being an excellent source of iron and zinc (8, 9). The BRS Xiquexique (white commercial subclass) has high iron (7.7 mg/100 g) and zinc (5.3 mg/100 g) contents that can minimize the deficiency of these micronutrients in the general population (10).

The bioavailability of minerals from foods is defined as the proportion of the minerals that can be absorbed and utilized within the body. The concept of bioavailability is associated not only with the absorption or uptake of nutrients by the intestinal mucosa, but its use also comprises transport, cellular uptake, and conversion of a nutrient into a biologically active form(s) (11).

On the contrary, bioaccessibility has been defined as the fraction of a compound that is released from its matrix in the gastrointestinal tract, and thus becomes available for intestinal absorption (12). *In vitro* studies of minerals in foods from plant sources consist of simulating the gastrointestinal digestion followed by measurement of mineral solubility (13–15). The study of iron and zinc bioaccessibility in different cowpea cultivars is important so that new varieties of this food can be introduced in the usual diet of the low-income population. Determination of iron and zinc bioaccessibility makes it possible to know their actual percentage of absorption, enabling the development of strategies to combat nutrition shortages in Brazil.

In a previous study, we evaluated zinc and iron contents in raw and cooked cowpea beans from five cultivars, before and after water soaking (16). Retention of these minerals was also measured. On the contrary, the present study aimed to analyze the influence of the same home-cooking techniques on the bioaccessibility of iron and zinc in cowpea cultivars. To accomplish this, after heat processing, an *in vitro* procedure using equilibrium dialysis during simulated gastrointestinal digestion was employed.

## Materials and methods

### Reagents

All glassware used in sample preparation and analyses were previously washed in water, immersed in a 5% nitric acid solution (1:1) for more than 1 h, and rinsed with ultrapure water (Milli-Q, Millipore, Milford, USA).

### Samples

The cowpea cultivars BRS Xiquexique, BRS Tumucumaque, BRS Aracê, BRS Guariba, and BR-17 Gurguéia were cultivated at Embrapa Mid-North, Teresina, Brazil. All the cultivars were grown in the same year in a field trial at Embrapa Mid-North, Teresina, Brazil (−5° 5′′S, 42° 48′′W), at an altitude of 72 m. The soil, an ultisol, was of sandy loam texture. Plowing, followed by disking, was carried out before sowing. Both pre-emergence (S-metolachlor) and post-emergence (glyphosate) herbicides were used for weed control. Crop cultivation was in 2010, in the dry growing season (June–August), using irrigation conditions of the sprinkler type (duration 2 h and irrigation interval of 5 days). The crops were manually harvested 75 days after sowing. The pods were dried in the sun, then machine threshed, and stored in plastic bags under refrigeration at 18°C.

### Cooking methods

To investigate the influence of heat processing on the zinc and iron bioaccessibility of the cowpea beans, two cooking methods were used: pressure cooking and cooking in a regular pan, with and without previous soaking in water. Samples of the five bean cultivars were cooked as follows: 1) in a regular pan without previous soaking, 2) in a regular pan after soaking, 3) in a pressure cooker without previous soaking, and 4) in a pressure cooker after soaking. The cooking times were previously established. The grains were boiled for approximately 30 and 10 min at 100°C, respectively, in a regular pan without and with prior water soaking. On the contrary, cooking was done for about 13 and 4 min at room temperature, respectively, in a pressure cooker without and with previous water soaking.

To cook the beans that were previously immersed in deionized water, a proportion of 100 g of beans to 400 mL of deionized water was used. After immersion in deionized water for 16 h at room temperature, in capped pans, beans were cooked according to the described methods. To cook the beans that were not previously immersed in deionized water, a proportion of 100 g of beans to 500 mL of deionized water was used. Cooking was carried out in Teflon-coated semi-capped pan and pressure cooker, with a capacity of 3 L each. Regular pan cooking was accomplished with a half-open lid; 500 mL of boiling water was added during cooking to replenish the loss of water that evaporated during cooking. No water replacement was necessary for the pressure cooking method. All experiments were performed in triplicate.

### Zinc and iron bioaccessibility

Zinc and iron bioaccessibilities in cowpea samples were determined based on an *in vitro* method described by Luten et al. (17), involving simulated gastrointestinal digestion with suitable modifications. All cowpea samples were finely ground in a stainless steel blender. Initially, for the gastric step, 10 g of the sample was weighed, ground in a stainless steel mixer, transferred to a 250 mL Erlenmeyer flask, and added to 80 mL of distilled water. Afterward, 3 ml of pepsin solution (P-7000) was added; the sample was cooled, homogenized, and incubated at 37°C, at pH 2.0 for 2 h, in a rotation of 100–120 strokes per minute. The pH was adjusted to 2.0 by adding 6 M HCl solution, and subsequently checked for any adjustments. A 20-ml aliquot of the pepsin digest was then mixed with 5 mL of pancreatin. Titratable acidity was measured in an aliquot of the gastric digest, by adjusting the pH to 7.5 with 0.2 mol/L sodium hydroxide in the presence of a pancreatin (P1750) – bile extract (B8631) mixture (4 g pancreatin+25 g bile extract+0.1 mol/L sodium bicarbonate). Titratable acidity was defined as the amount of 0.2 mol/L sodium hydroxide required to reach pH 7.5. To simulate intestinal digestion, segments of dialysis tubing (molecular mass cut off of 10 kDa) containing 25 mL of a sodium bicarbonate solution, equivalent in moles to the amount of sodium hydroxide needed to neutralize the product of the gastric digestion (titratable acidity determined as above), were placed in Erlenmeyer flasks containing the gastric digest. The flasks were incubated at 37°C for 30 min or longer with shaking, until the pH of the digest reached 5.0. Five milliliters of the pancreatin–bile extract mixture were then added and incubation was continued for 2 h.

At the end of the simulated gastrointestinal digestion, zinc and iron present in the dialysate were analyzed by ICP (inductively coupled plasma) atomic emission spectrometry (Spectro Analytical Instruments – Spectroflame model P, Perkin Elmer, Waltham, Massachusetts, USA) (18). The dialysate portion of the total mineral present in the sample (expressed as percent) represented the bioaccessible mineral.

Bioaccessibility (%) was calculated as follows:

$$\text{Bioaccessibility}(\%)=100\times \frac{Y}{Z}$$

where Y is the element content of the bioaccessible fraction (mg mineral element/100 g grain), and Z is the total zinc or iron content (mg mineral element/100 g grain).

### Analysis of Fe and Zn in cowpea dialysates

Digestion and quantification of Fe and Zn in samples of cowpea dialysates were as described in the Standard Operating Procedures (SOP's) of the Laboratory of Minerals of Embrapa Food Technology, certified by NBR/ISO 17025:2005.

- Mineralization by microwave cavity – POP MIN 192 – 008, rev. 06;

- Quantification: ICP – OES – POP MIN – 026, rev. 00.

About 5 g of sample was weighed in polyethylene tubes for rotor type Xpress, and 6 mL of nitric acid PA ACS ISO was added. The samples were digested in a microwave cavity, MARS5 model (CEM, Matthews, North Carolina, USA), with a maximum power of 1,600 W for 15 min. The digest was transferred quantitatively into a 25-mL volumetric flask, completing the volume with ultrapure water.

Quantification of Fe and Zn was performed by optical emission spectrometry with inductively coupled plasma (ICP-OES), model Optima 2100DV (Perkin Elmer, Waltham, Massachusetts, USA), with concentric nebulizer and cyclonic mist chamber with optics and sequential viewing torch radial and axial. Table 1 shows the condition of the equipment and the method used for quantification.

**Table 1:** Experimental conditions and quantification method

Operating conditions									
Potency RF (W)					1,300				
Nebulizer flow (L min^−1^)					0.80				
Plasma flow (L min^−1^)					15				
Sample flow (L min^−1^)					1.50				
Nebulizer					Concentric MEINHARD^®^ Type C				
Mist chamber					Cyclonic (glass) MEINHARD^®^				
			Points of the curve						
				
Element	Wavelength (nm)	View	00	01	02	03	04	05	Unit
Fe	259.939	Axial	0	5	10	20	50	100	µg/kg (ppb)
Zn	213.857	Axial	0	50	100	200	500	1,000	µg/kg (ppb)

### Statistical analysis

Results were expressed as means±standard deviations of three separate determinations. All data were treated by analysis of variance (ANOVA). Comparison among the treatment averages was by the least significance difference test (LSD) at a level of 5% of probability. All of the statistical analyses were carried out using Statistica software version 5.1.

## Results

The iron bioaccessibility of cowpea varieties in the raw beans after cooking by different methods can be found on Table 2. BRS Xiquexique showed the best percentage of iron bioaccessibility (4.37%) in raw beans.

**Table 2:** Iron bioaccessibility levels in raw and cooked beans after different cooking methods of five cowpea cultivars

	Bioaccessible iron (%)				
	
	Pressure cooker			Regular pan	
		
Cultivars	RAW	Without soaking water	With soaking water	Without soaking water	With soaking water
BRS Xiquexique	4.37^a^±0.97	44.33^aA^±1.07	8.86^aB^±2.66	2.94^aA^±1.16	6.78^aB^±0.19
BRS Tumucumaque	3.30^a^±0.01	34.94^bB^±0.44	21.57^bC^±2.56	5.84^bC^±0.74	5.65^aC^±1.70
BRS Aracê	2.62^b^±0.11	14.84^cD^±0.79	6.46^aE^±0.80	6.91^bD^±0.08	2.40^bE^±0.16
BRS Guariba	2.59^b^±0.19	25.75^dF^±0.53	40.68^cG^±4.12	4.72^cF^±0.01	6.83^aG^±0.36
BR-17 Gurguéia	2.50^b^±0.27	8.61^eH^±0.48	9.83^aH^±5.11	8.72^dH^±0.02	8.92^cH^±1.27

Different letters within the same column differ significantly at 5% probability. Different uppercase letters within the same line differ significantly at 5% probability (ANOVA, *p*<0.05). Values are expressed as average ±SEM of three independent determinations.

Cooking in a pressure cooker without previous soaking resulted in iron bioaccessibility values that varied from 44.33% (BRS Xiquexique) to 8.61% (BR-17 Gurguéia). In addition, samples cooked after soaking revealed bioaccessibility values from 21.57% (BRS Tumucumaque) to 6.46% (BRS Aracê). Thus, cooking in a pressure cooker without previous soaking resulted in the highest percentage of iron bioaccessibility (44.33%). However, there was a reduction in the percentage of iron bioaccessibility in all cultivars processed by boiling in a pressure cooker after soaking, except for cultivar BRS Guariba. This can be attributed to the higher iron content present in the raw grain of cultivar BRS Guariba (16).

Evaluation of iron bioaccessibility of cultivar BRS Xiquexique shows that it responded better on cooking without previous soaking.

Iron bioaccessibility varied from 8.72% (BR-17 Gurguéia) to 2.94% (BRS Xiquexique) when beans were cooked in a regular pan without prior soaking. In addition, the samples cooked after soaking revealed values from 8.92% (BR-17 Gurguéia) to 2.40% (BRS Aracê). Cultivar BR-17 Gurguéia had the highest percentage of iron bioaccessibility when cooked in a regular pan either with or without previous soaking.

The results of zinc bioaccessibility in cowpea cultivars after different methods of cooking can be seen in Table 3. Zinc bioaccessibility varied from 56.32% (BRS Xiquexique) to 37.52% (BRS Tumucumaque) when beans were cooked in the pressure cooker without previous soaking. In addition, the samples cooked after soaking revealed zinc bioaccessibility ranging from 51.64% (BRS Guariba) to 18.96% (BRS Xiquexique). For this thermal process, soaking did not significantly affect (*P<*0.05) zinc bioaccessibility, except in samples from cultivar BRS Xiquexique.

**Table 3:** Zinc bioaccessibility levels in raw and cooked beans after different cooking methods of five cowpea cultivars

	Bioaccessible zinc (%)				
	
	Pressure cooking			Regular pan	
		
Cultivars	RAW	Without soaking water	With soaking water	Without soaking water	With soaking water
BRS Xiquexique	37.43^a^±2.30	56.32^aA^±3.19	18.96^aB^±2.72	42.75^aA^±2.60	45.12^aA^±4.92
BRS Tumucumaque	39.50^a^±0.79	37.52^bC^±1.49	40.50^bC^±0.70	38.81^aB^±3.78	45.91^aC^±1.13
BRS Aracê	38.62^a^±0.28	44.70^aD^±2.46	44.68^bD^±2.04	42.81^aD^±2.34	31.01^bE^±2.08
BRS Guariba	40.82^b^±2.01	47.65^aE^±4.73	51.64^cE^±3.11	52.78^bF^±5.22	40.90^cG^±2.42
BR-17 Gurguéia	37.71^a^±0.96	52.48^cF^±3.23	51.31^cF^±1.32	43.46^aH^±6.43	37.10^bI^±4.52

Different letters within the same column differ significantly at 5% probability. Different uppercase letters within the same line differ significantly at 5% probability (ANOVA, *p*<0.05). Values are expressed as average ±SEM of three independent determinations.

Cultivar BRS Xiquexique presented a lower percentage of zinc when processed in a pressure cooker with prior immersion, while the values for the other cultivars were nearly equal. This result can be attributed to the lower content of zinc present in the raw grain of cultivar BRS Xiquexique (16).

When samples were cooked in a regular pan without prior soaking, zinc bioaccessibility varied from 52.78% (BRS Guariba) to 38.81% (BRS Tumucumaque), while cooking after soaking led to values ranging from 45.91% (BRS Tumucumaque) to 31.01% (BRS Aracê). Therefore, cultivar BRS Guariba had the highest percentage of bioaccessible zinc (52.78%) after cooking in a regular pan without previous soaking. However, soaking the samples prior to cooking in a regular pan significantly reduced (*P*<0.05) zinc bioaccessibility in cultivars BRS Aracê, BRS Guariba, and BR-17 Gurguéia.

## Discussion

Hemalatha et al. (15) examined the influence of processing on iron bioaccessibility in cowpea beans and found iron bioaccessibility of 3.98% after cooking in a pressure cooker, whereas in our study the highest percentage of iron bioaccessibility was 34.94%. A possible explanation for the difference in results compared to our studies is the fact that the authors failed to mention the mode of cooking, cooking time, or whether water immersion was used to process the grains. Of the two heat-processing methods employed in our study, pressure cooking is the commonly adopted practice in Brazilian households.

On the contrary, bioavailability of iron is known to be influenced by various dietary components, which include inhibitors of absorption, such as phytic acid, tannins, dietary fiber, and calcium (19). Most dietary iron is in the form Fe3 +, which is reduced to Fe2+by ascorbic acid in gastric secretions or ferrireductase in the mucosa, and then absorbed. Anything that prevents this reduction decreases the absorption of iron, for example, the production of an insoluble complex with other dietary compounds such as tannin (present in tea), phytate, and certain fibers (20).

Four different colored beans (white, red, pinto, and black beans) were investigated for factors affecting iron bioavailability. The results show that white beans contained higher levels of bioavailable iron compared with red, pinto, and black beans. These differences in bioavailable iron were not due to bean-iron and bean-phytate concentrations. Flavonoids in the colored bean hulls were found to be contributing to the low bioavailability of iron in the non-white-colored beans (21). The phenolic compounds such as phenolic acids, flavonoids, and tannins are potent inhibitors of iron absorption and are present in plant foods (particularly found in legumes film and whole grains). The polyphenols may react with divalent metal ions, such as iron, through their carboxyl and hydroxyl groups, forming insoluble and stable complexes in the intestinal lumen, thus reducing the absorption of non-heme iron (22).

According to Bassinello (23), retention of important minerals such as iron and zinc in beans can be influenced by the use of water immersion during cooking. The minerals lost in the cooked grain are present in the cooking water, and the broth can contain up to 73% of mineral from the raw grain, depending on the cultivar and mineral. On the contrary, studies by Rodrigues et al. (24) indicate that immersion can reduce the content of minerals that can be partially or completely eliminated by solubilization and removal of the water dip or may be retained in the cooking broth. Lestienne et al. (25) showed that soaking on its own was not a good method to improve mineral bioavailability. However, their results showed that in combination with other treatments, or with optimized soaking conditions, it could be useful.

Most of the studies available on the analysis of beans are from other countries where beans are prepared for consumption in a different way than in Brazil, that is, with whole grains and broth. Thus, there is a gap in applying those findings to the Brazilian reality, coupled to a scarcity of studies, since the main losses of micronutrients occur during the cooking process and through dilution in the broth (26). To the best of our knowledge, the present study is the first in which iron and zinc bioaccessibility was determined in cowpea beans of conventionally bred cultivars processed by home-cooking methods.

In developing countries, children aged above 2 years do not consume foods rich in iron content (liver, meat, and fish) in sufficient quantities. Thus, it is essential to determine the bioaccessibility of minerals such as iron and zinc to know their actual percentage of absorption.

Hemalatha et al. (14, 15) found 53% of zinc bioaccessibility in the raw beans of cowpea. They obtained 41.50% of zinc bioaccessibility after cooking samples in a pressure cooker, whereas in our study a value of 56.32% was seen (BRS Xiquexique). Nevertheless, zinc bioaccessibility in raw cowpea was higher than iron bioaccessibility in our studies. According to Raes et al. (27), the lower leaching out of zinc compared with iron can be attributed to the fact that these minerals are not linked to the same molecules (zinc is highly associated with enzymes and proteins) and that they are not located at the same place in the seeds. According to Naozuka and Oliveira (28), these minerals are complexed to other constituents, possibly proteins, and the types of bonds and associated constituents could be different for zinc and iron. According to Lombardi-Boccia et al. (29), heat processing of food generally alters the bioavailability of nutrients. Digestibility and hence absorption of micronutrients such as iron is believed to be improved upon heat processing, which results in softening of the food matrix, with release of protein-bound iron, thus facilitating its absorption. The differences in the percentages of zinc and iron bioaccessibility in cowpea beans can be attributed to the different chemical forms of these metals and their possible different localizations in the beans.

The increase in zinc bioaccessibility upon heat treatment observed in the present study could be attributed to interactions of zinc with proteins, and/or other bean constituents, thereby hindering its absorption. Nevertheless, zinc bioaccessibility in raw cowpea was higher than iron bioaccessibility in our studies.

Strategic breeding of germplasm and transgenic methods have increased the density of several essential micronutrients in staple food crops (30). Micronutrients primarily targeted for increased concentrations in plant foods are iron and zinc, as these continue to be the major nutritional deficiencies in developing countries. The diets of populations at risk of food insecurity consist largely of rice, beans, wheat, and potato, which lack adequate amounts of these micronutrients (31).

## Conclusions

Heat treatment of cowpea cultivars produced contrasting effects on zinc and iron bioaccessibility. In all cultivars, higher percentages of zinc compared with iron bioaccessibility were found after cowpea beans were cooked in a pressure cooker and in a regular pan. On the contrary, iron bioaccessibility values suggest that cooking in a regular pan did not have a good effect on iron availability, since the percentage of bioaccessible iron was lower than zinc. Consequently, determination of iron and zinc bioaccessibility makes it possible to find out the actual percentage of absorption of such minerals and allows the development of efficient strategies for low-income groups to have access to foods with high levels of these micronutrients.
